# Effect of flame retardants on mechanical and thermal properties of bio-based polyurethane rigid foams

**DOI:** 10.1039/d1ra05519d

**Published:** 2021-09-16

**Authors:** Qirui Gong, Liangyu Qin, Liangmin Yang, Keke Liang, Niangui Wang

**Affiliations:** College of Chemistry and Chemical Engineering, Hubei University Wuhan 430062 China nianguiwang@hubu.edu.cn

## Abstract

A soy oil-based polyol (HSBP) was synthesized from epoxidized soy oil through a ring-opening reaction with distilled water. A phosphorus-containing flame retardant (DOPO–HSBP) was synthesized through the reaction of 9,10-dihydro-9-oxa-10-phosphaphenanthrene-10-oxide (DOPO) and HSBP. A nitrogen-containing flame retardant (T–D) was prepared by the reaction of diethanolamine with glycol diglycidyl ether. The structures of HSBP, DOPO–HSBP, and T–D were characterized by Fourier transform infrared spectroscopy (FT-IR) and nuclear magnetic resonance (^1^H NMR). The flame-retardant rigid polyurethane foam (PPUFs and NPUFs) was prepared successfully by mixing HSBP, DOPO–HSBP, and T–D. The effects of DOPO–HSBP content on the mechanical, thermal, and flame-retardant properties of PPUFs and NPUFs were investigated by tensile tests, thermogravimetric analyses (TGA), limiting oxygen index (LOI), and UL-94 vertical burning level. The morphology of PPUFs and NPUFs was studied *via* scanning electron microscopy (SEM). With the increase in the percentage of DOPO–HSBP added, the flame retardant property of rigid polyurethane foam (RPUF) was greatly improved. When the phosphorus-containing flame retardant DOPO–HSBP was added to 50% of the RPUF with the nitrogen-containing flame retardant T–D, the LOI value of the foam increased from 18.3 to 25.5, and the UL-94 result was classified as “V-0” with almost no effect on the mechanical properties of the RPUF. The results showed that the phosphorus and nitrogen synergistic flame retardants of DOPO–HSBP and T–D can endow excellent flame retardant properties to RPUF without affecting its mechanical properties.

## Abstract 2

A vegetable oil-based polyurethane rigid foam containing a phosphorus–nitrogen dualflame retardant system was prepared, and the foam exhibited not only excellent flame retardant properties but also good mechanical properties.

## Graphical abstract

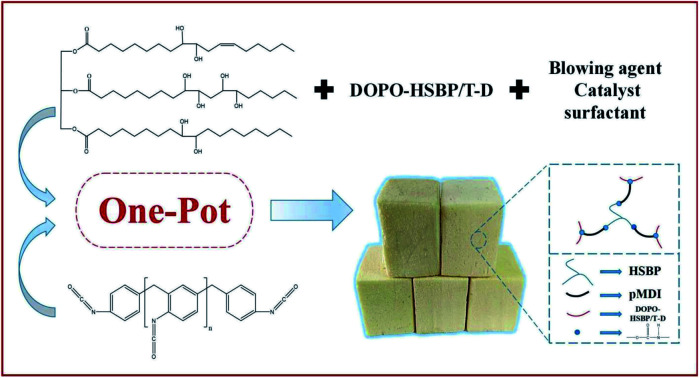

## Introduction

Rigid polyurethane foam (RPUF) is an economical and efficient energy-saving material that is widely used in fields of furniture, transportation, construction materials, refrigerator insulation because of its light weight, high strength, and excellent mechanical and thermal insulation properties.^1–4^ However, its porous structure makes it easy to be ignited and burn rapidly after being exposed to fire, releasing large amounts of heat and smoke, leading to serious fire accidents.^5,6^ This limits the application of rigid polyurethane foam in many fields. Therefore, the study of flame retardant rigid polyurethane foams is of great importance.

In order to improve the flame retardancy of RPUF, adding flame retardants to RPUF would be effective.^6^ There are two main approaches to add flame retardants to RPUF: additive-type flame retardants and reactive-type flame retardants.^7,8^ Examples of additive flame retardants include expandable graphite (EG),^9,10^ ammonium polyphosphate (APP),^11^ melamine and its derivatives,^12,13^ inorganic phosphorus-containing compounds,^14^*etc.* However, they are gradually replaced by another way due to their poor compatibility, the large negative impact on the mechanical properties, and the easy leaching of the RPUF.^15^ Reactive flame retardants are more stable in RPUF, and the flame retardant element P/N is introduced into the main chain of RPUF by participating in the foaming reaction to achieve the flame retardant effect.^16–19^ DOPO and its derivatives are some of the reactive flame retardants that have attracted much attention in recent years. Compared with other straight-chain small molecule flame retardants, DOPO and its derivatives have better stability due to their aromatic structure.^20,21^ For instance, in the work of Wang *et al.*, a bifunctional flame retardant (PDEP) based on DOPO and phosphate was synthesized, and the LOI of RPUF was increased from 18.5 to 22.9 by the addition of this flame retardant.^22^ However, a single flame retardant system can only improve the flame retardancy of the foam to a very limited extent. Some studies indicate that the synergistic effect of phosphorus and nitrogen can greatly improve its flame retardant efficiency.^23^

In addition to the flame retardancy of rigid polyurethane foams, biodegradability has also been a major concern for environmental protection and sustainable development in recent years. The adoption of bio-based polyols to prepare RPUF would be a good answer for those concerns.^24^ Vegetable oil can be used to produce bio-based RPUF due to the presence of many reactive sites on its aliphatic chain. For example, Veronese *et al.* synthesized RPUFs by using soybean oil or castor oil.^25^ Guo *et al.* prepared soy-based polyol from epoxidized soy oil ring-opened by methanol and the resulting RPUF exhibited comparable mechanical and insulating properties to other foams from petrochemical feedstocks.^26^ Ji *et al.* synthesized different soy-based polyols by reacting epoxidized soy oil with methanol, phenol, and cyclohexanol. At 25 wt% of soy-based polyol, the introduction of phenol can improve the mechanical and thermal properties of the foam.^27^

The purpose of this work is to synthesize soybean oil-based polyol as a raw material for RPUF, a polyol DOPO–HSBP as a P-containing flame retardant, and polyol T–D as an N-containing flame retardant. The effects of the content of DOPO–HSBP and the phosphorus–nitrogen synergistic effect of T–D addition on the mechanical properties and flame retardant properties of RPUF were analyzed using the mechanical test, scanning electron microscopy (SEM), thermogravimetric analysis (TGA), LOI, and UL-94. Finally, the RPUFs with greatly improved flame retardancy and almost no effect on mechanical properties were successfully synthesized.

## Experimental section

### Materials

Epoxidized soybean oil (ESO, epoxy content: 6.4 wt%) was prepared using the method of Klaas.^28^ Soy oil-based polyol (HSBP) was prepared by a ring-opening reaction of epoxidized soybean oil by distilled water using a procedure previously reported.^29–31^ 9,10-Dihydro-9-oxa-10-phosphaphenanthrene-10-oxide (DOPO) was supplied by Guangdong Weng Jiang Chemical Co., Ltd. (Guangdong, China). Glycol diglycidyl ether was purchased from Changzhou Runxiang Chemical Co., Ltd.; China. Diethanolamine was obtained from Sinopharm Chemical Reagent Co., Ltd. Polymeric 4,4′-diphenylmethane diisocyanate (pMDI, NCO content: 30.87 wt%) was obtained from Wanhua Chemical Group Co., Ltd., China. Surfactant (AK-8805) was supplied by Jiangsu Maysta Chemical Co., Ltd., China. The catalyst (dibutyltin dilaurate, DBTDL) was supplied by Tianjin Dingshengxin Chemical Industry Co., Ltd., China. Distilled water was used as a blowing agent.

### Preparation of DOPO–HSBP

**Scheme 1:**
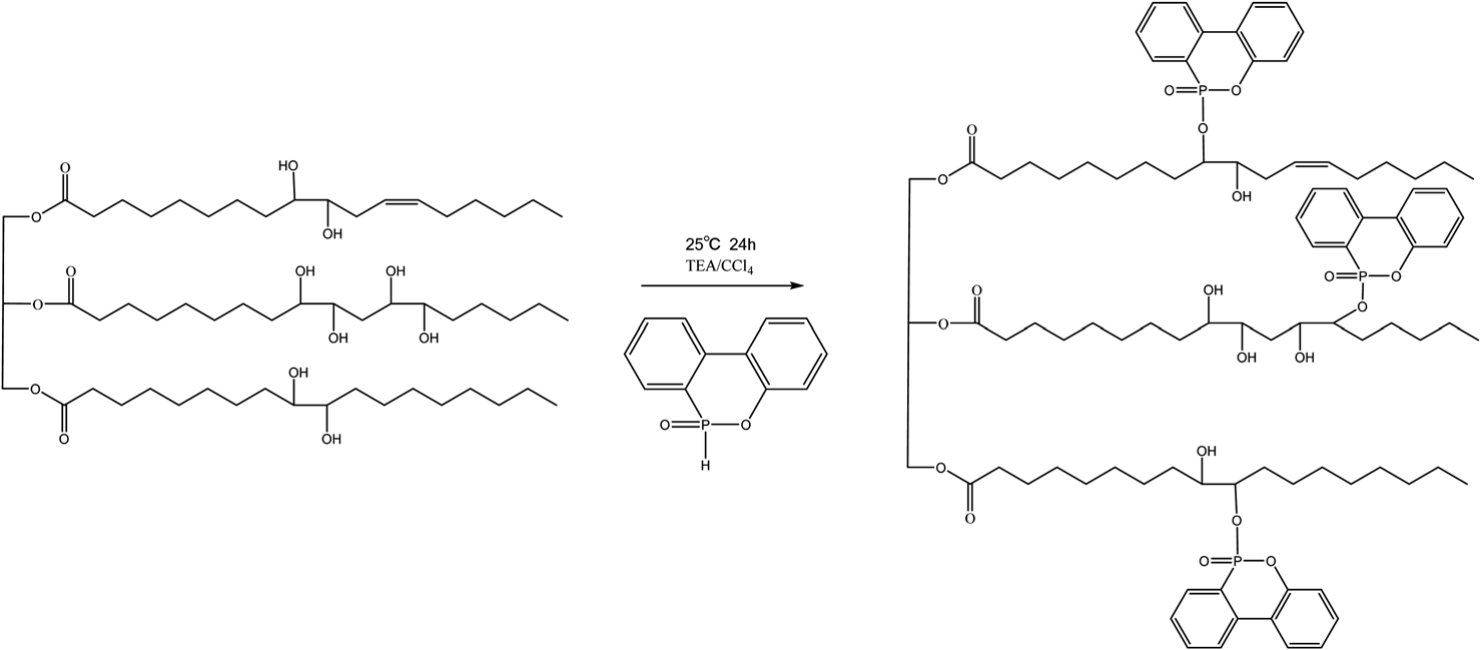
The preparation route of DOPO–HSBP.

The DOPO–HSBP was prepared by reacting DOPO with HSBP. During a process, 40 g HSBP, 25.8 g DOPO, 12.5 g TEA, and 40 g dichloromethane were added to a round-bottomed three-necked flask equipped with a mechanical stick. Carbon tetrachloride was added dropwise to the reaction mixture at ice bath over 30 minutes. The temperature of the reaction was maintained at 25 °C for 24 h. After the reaction completion, the reaction mixture was extracted with ethyl acetate and washed with deionized water. Ethyl acetate and deionized water were removed by rotary evaporator. The preparation route of DOPO–HSBP was shown in Scheme 1. The properties of the HSBP and DOPO–HSBP were listed in Table 1.

**Table 1:** The properties of HSBP and DOPO–HSBP

Polyol	Hydroxyl number (mg KOH per g)	Acid number (mg KOH per g)	Viscosity (mPa s) (at 25 °C)	Color
HSBP	420	2.2	50 638	Pale yellow
DOPO–HSBP	120	4.3	72 382	Pale yellow

### Preparation of T–D

**Scheme 2:**
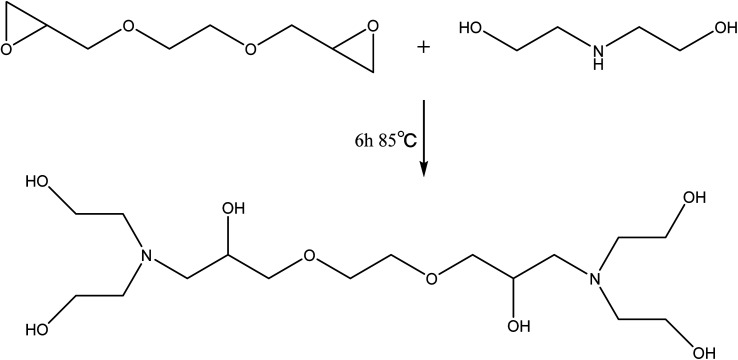
The preparation route of T–D.

Diethanolamine (54 g) and glycol diglycidyl ether (62 g) were charged into a round-bottomed three-necked flask equipped with a mechanical stick and a thermometer. The temperature of the reaction was maintained at 85 °C for 6 h. After the reaction completion, the resulting product can be used for the next step without any treatment. The preparation route of T–D was shown in Scheme 2.

### Preparation of rigid polyurethane foams (RPUFs)

**Scheme 3:**
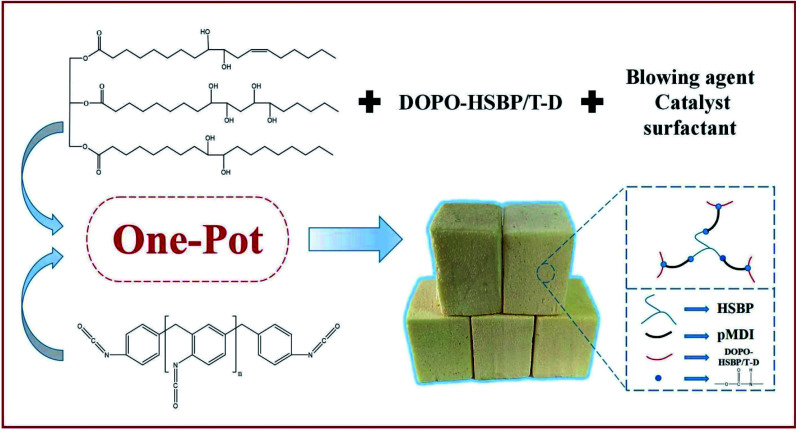
The preparation route and structure of RPUFs.

The RPUFs were prepared using a free-rise method according to the formulation as shown in Table 2. The –NCO/–OH ratio of the systems was set as 1.1. The polyol blends were mixed with surfactant (AK8805), catalyst (dibutyltin dilaurate), and blowing agent (distilled water), in the required proportions under ambient conditions for approximately 120 s. The pMDI was added quickly into the mixture and mixed for another 15 s. Finally, the mixture was immediately poured into an open mold to produce free-rise foam. The obtained RPUFs were completely cured at room temperature for 7 days before analysis. The preparation route and structure of RPUFs are shown in Scheme 3.

**Table 2:** The formulations of RPUFs

Sample	HSBP content (wt%)	DOPO–HSBP content (wt%)	T–D content (wt%)	AK8805 content (wt%)	DBTDL content (wt%)	H_2_O content (wt%)	Isocyanate index [*n*(–NCO)/*n*(–OH)]
PPUF-0	100	0	0	3	1	1.5	1.1
PPUF-10	90	10	0	3	1	1.5	1.1
PPUF-20	80	20	0	3	1	1.5	1.1
PPUF-30	70	30	0	3	1	1.5	1.1
PPUF-40	60	40	0	3	1	1.5	1.1
PPUF-50	50	50	0	3	1	1.5	1.1
NPUF-0	90	0	10	3	1	1.5	1.1
NPUF-10	80	10	10	3	1	1.5	1.1
NPUF-20	70	20	10	3	1	1.5	1.1
NPUF-30	60	30	10	3	1	1.5	1.1
NPUF-40	50	40	10	3	1	1.5	1.1
NPUF-50	40	50	10	3	1	1.5	1.1

### Characterization

FT-IR spectra were measured by Spectrum One PerkinElmer Fourier transform infrared spectrometer (PerkinElmer Co., American). The FT-IR spectra were collected using 64 scans in the wavenumber range of 4000–400 cm^−1^ with a resolution of 4 cm^−1^.

^1^H NMR spectra were tested by Varian Inova 600 nuclear magnetic resonance spectrometer (American Varian Co., American) at room temperature, using tetramethylsilane (TMS) as a reference, CDCl_3_ as solvent for sample.

The density of RPUF samples was measured according to the American Society of Testing Materials (ASTM) designation: D162208 (2008). The average values of five samples were recorded.

The compressive strength of foams was recorded on a CMT4000 universal testing machine according to (Shengzhen, China) according to ASTM designation: D1621-10. Each sample used for the test was 50 × 50 × 50 mm^3^ (length × width × height). At least five samples were tested to obtain average values in mechanical tests.

A NETZSCH 209F1 TA Instruments was employed for thermogravimetric analysis (TGA), and the RPUF samples were heated to 750 °C at a heating rate of 10 °C min^−1^ under a dynamic nitrogen flow of 50 mL min^−1^.

The morphologies of the foams were analyzed with a JSM-6510LV scanning electron microscope (SEM) operating at 20 kV, and the samples were sputter-coated with a thin layer of gold. The scanning electron microscopy was measured along parallel foam rise direction.

Limiting oxygen index (LOI) values were measured using an LOI Analyzer instrument according to the ASTM D2863-97. The dimensions of the samples were 127 × 10 × 10 mm^3^ (length × width × thickness). UL-94 vertical burning test was recorded using a CFZ-2-type vertical burning tester (Jiangning Analysis Instrument Co., Nanjing, China) according to ASTM D3801-96. The dimensions of the samples were 127 × 13 × 10 mm^3^ (length × width × thickness).

## Results and discussion

### FT-IR spectra of HSBP, DOPO–HSBP, DEA, TMA, and T–D

**Fig. 1:**
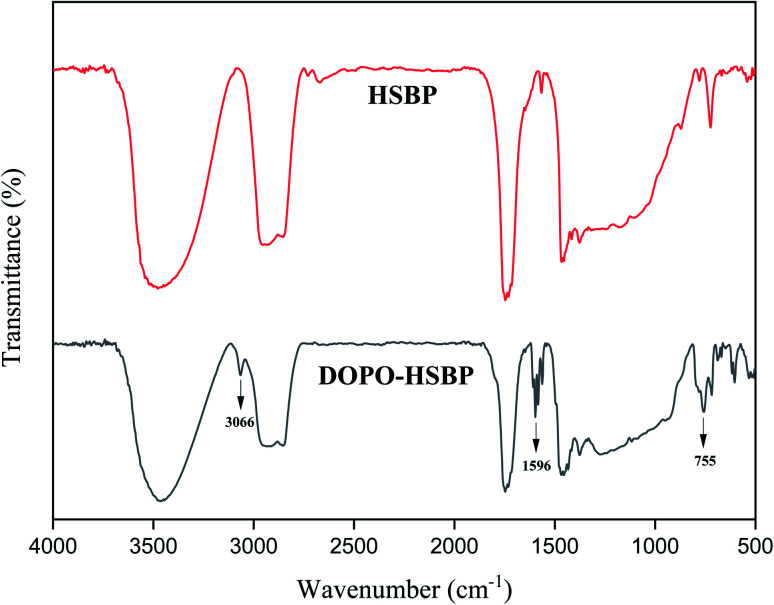
FT-IR spectra of HSBP and DOPO–HSBP.

**Fig. 2:**
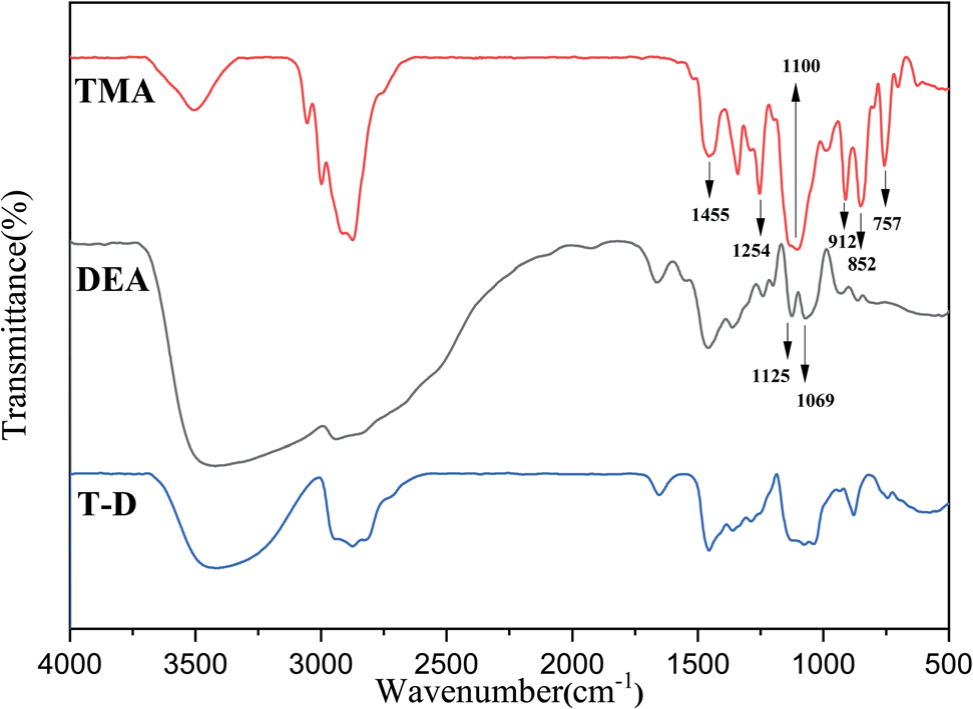
FT-IR spectra of TMA, DEA, and T–D.

The FTIR spectra of HSBP and DOPO–HSBP are shown in Fig. 1. The aromatic C–H absorption around 3066 cm^−1^.^32^ The absorption around 755 cm^−1^ and 1596 cm^−1^ corresponds to vibration with Ph–P and C=C stretching in aromatic functional groups respectively.^33,34^ The absent peak at 2385 cm^−1^ (P–H) indicated that the reaction of HSBP and DOPO has done.^35^Fig. 2 shows the FTIR of TMA, DEA, and T–D. It can be observed from the spectrum of TMA that the peak at 912 cm^−1^, 852 cm^−1^, 1254 cm^−1^, and 757 cm^−1^ corresponds to epoxy bond characterized absorbing. In the FTIR spectra of T–D, the disappearance of the epoxy absorption peak. The stretching vibration peak at 1100 cm^−1^ and the deformation vibration peak at 1455 cm^−1^ in TMA corresponds to C–O–C and –CH_2_ respectively still exist. The characterized absorbing peak at 1125 cm^−1^ and 1069 cm^−1^ in DEA corresponds to C–N and C–O respectively still exists, which indicates that DEA and TMA have a ring-opening reaction. All these verified the successful synthesis of DOPO–HSBP and T–D. The chemical structures of DOPO–HSBP and T–D are further confirmed by ^1^H NMR.

### ^1^H NMR analysis of HSBP, DOPO–HSBP, DEA, TMA, and T–D

**Fig. 3:**
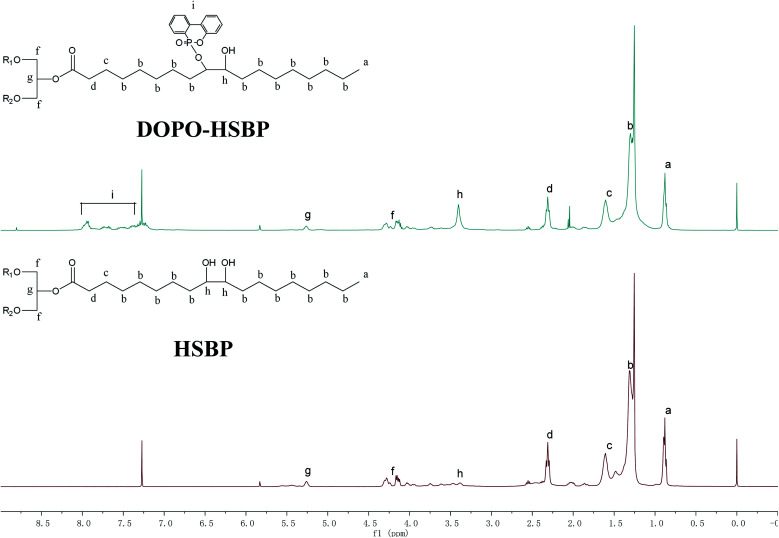
^1^H NMR spectrum of HSBP and DOPO–HSBP.

**Fig. 4:**
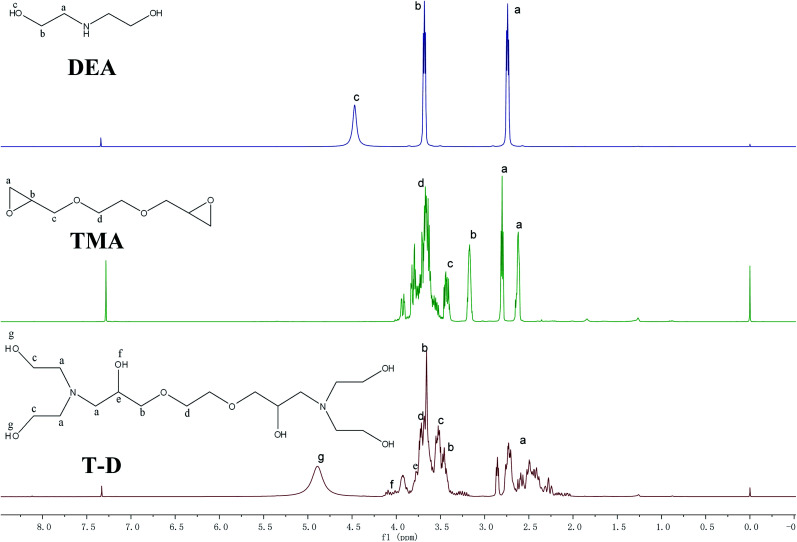
^1^H NMR spectrum of TMA, DEA, and T–D.

Fig. 3 shows ^1^H NMR spectra of HSBP and DOPO–HSBP that reveals the Ar–H (peak i) peaks at 7.03–8.23 ppm, and the peak related with P–H of DOPO at 9.0 ppm disappears in the spectra of DOPO–HSBP.^36,37^ All the above indicated the successful synthesis of DOPO–HSBP. Fig. 4 compares the ^1^H NMR spectrum of T–D with that of TMA. We can see that a new peak is observed at 4.89 ppm (peak g). Furthermore, the peak related to CH–O–C of TMA at 3.17 ppm (peak b) disappears in the spectra of T–D. All the aforementioned results suggested the successful synthesis of T–D.

### Apparent density

**Fig. 5:**
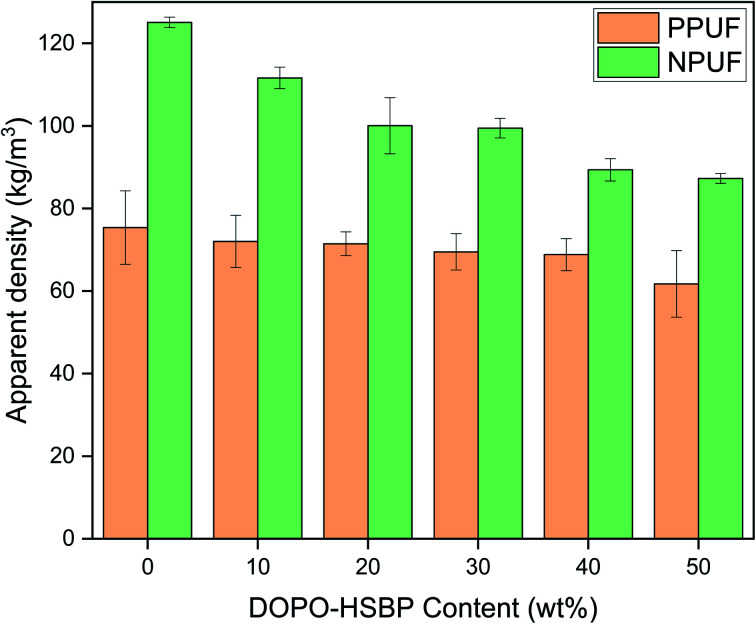
Apparent density of PPUFs and NPUFs.

The apparent density of PPUFs and NPUFs prepared from blends with different ratios of DOPO–HSBP is shown in Fig. 5. The relevant literature shows that the apparent density of rigid polyurethane foam is closely related to the compressive strength.^38^ It can be observed from Fig. 5 that the apparent density of both PPUFs and NPUFs tends to decrease with the increase of DOPO–HSBP content. When the content of DOPO–HSBP increased from 0 to 50%, the apparent densities of PPUFs and NPUFs decreased by 18% and 30%, respectively. These changes could be attributed to the decrease in crosslink density of the foam due to the addition of DOPO–HSBP. However, it is not difficult to find that the apparent density of NPUF-50 is still higher than that of neat RPUF. This indicates that the moderate addition of T–D and DOPO–HSBP has almost no effect on the physical properties of the foams.

### Microphotographs

**Fig. 6:**
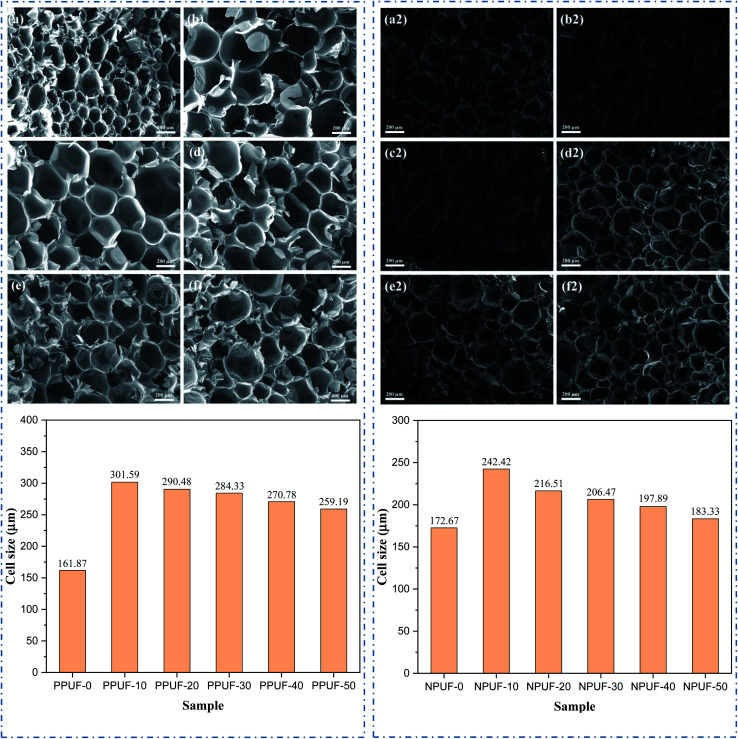
SEM images and cell size of (a) PPUF-0, (b) PPUF-10, (c) PPUF-20, (d) PPUF-30, (e) PPUF-40, (f) PPUF-50, (a2) NPUF-0, (b2) NPUF-10, (c2) NPUF-20, (d2) NPUF-30, (e2) NPUF-40 and (f2) NPUF-50.

The morphology changes of PPUFs and NPUFs are shown in Fig. 6. A typical RPUF structure was observed in Fig. 6, with open and closed pores of the sphere or polygonal shape. By adding phosphorus-containing flame retardant DOPO–HSBP, it can be seen that there is a significant increase in the cell size compared with the PPUF-0, but with the further increase of DOPO–HSBP content from 10% to 50%, the cell size of the foam is reduced by 14%. This may be due to the decrease in the hydroxyl value of DOPO–HSBP compared to HSBP, which leads to a decrease in the cross-link density of the foam, a decrease in the number of pores, and an increase in the cell sizes. When the content of DOPO–HSBP increases further, the cell growth in the foaming process is weakened due to its higher viscosity, which eventually leads to smaller pores. On the other hand, it can be clearly observed that when the N-containing flame retardant T–D is added, there is a slight increase in the foam pore size compared to the foam without the flame retardant added. Comparing PPUF and NPUF, we can see that when T–D is added, the pore size still increases and then decreases with the increase of DOPO–HSBP content, but the cell size of NPUF-50 has been reduced to a size close to RPUF-0. This may be due to the high hydroxyl value and low viscosity of T–D. This means that NPUF-50 has little effect on the foam cell size compared with the RPUF-0, and the foam can still retain its original excellent properties such as thermal insulation.

### Mechanical properties

**Fig. 7:**
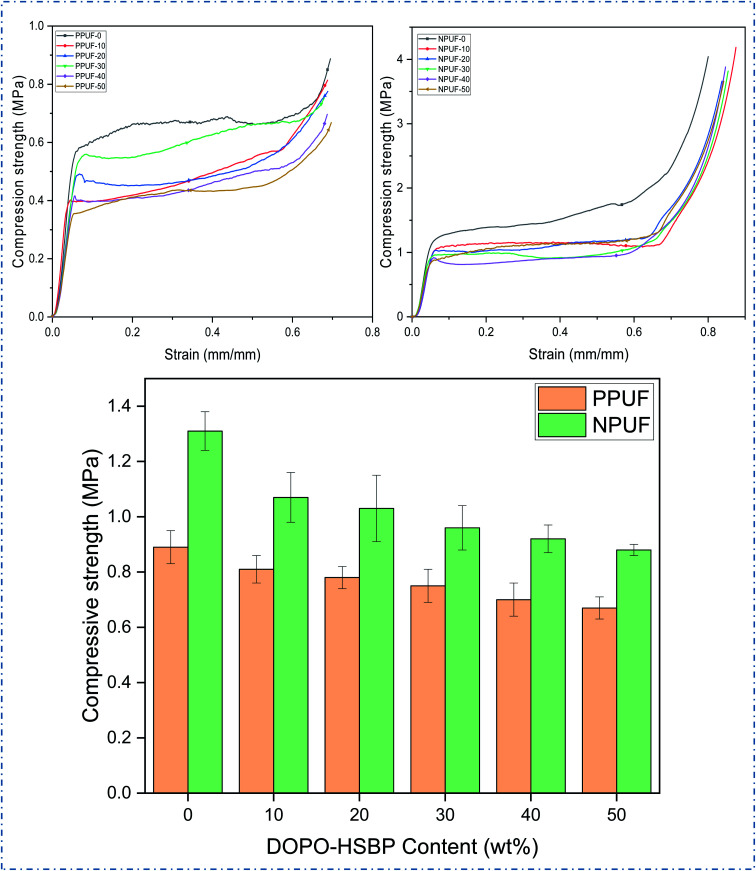
Compressive stress–strain curves and compression strength of PPUFs and NPUFs.

Fig. 7 shows the stress–strain curves of PPUFs and NPUFs prepared from different ratios of DOPO–HSBP and the compression strength (10% compression strain) of PPUFs and NPUFs are listed in Table 3. At low strains, the foam exhibited linear-elastic deformation, followed by a continuous deformation platform, which may be the result of brittle fracture of the pore structure of the polyurethane foam, and at high strains, the densification of the foam caused the polymer to harden, making stress continues to increase.^39^ It was reported in the literature that the mechanical properties of RPUFs are closely related to the hydroxyl value of the polyol, the size of the foam pores, and the density of the foam.^40^ As shown in Table 3, with increasing the DOPO–HSBP content, the compressive strength of the corresponding foam decreased from 0.89 MPa to 0.67 MPa. With the addition of T–D, the compressive strength of NPUFs has increased significantly compared to PPUFs. Although the overall compressive strength of the foam still tends to decrease with the increase of DOPO–HSBP, the compressive strength of the foam can still reach 0.88 MPa (comparable to the compressive strength of the foam without flame retardants) even when the maximum amount of DOPO–HSBP is added to 50%. The reason for this phenomenon may be due to the lower hydroxyl value of DOPO–HSBP (compared with HSBP), which leads to a lower cross-link density of the foams, thus resulting in larger foam pores and ultimately a reduction in the compressive strength of the foams. The addition of T–D greatly compensates for this shortcoming, as its higher hydroxyl value greatly increases the crosslink density of the foams and acts as a hard segment during synthesis, increasing the composition of the hard segment of the foams. This leads to an overall increase in the compressive strength of the foams. According to GB T 21558-2008, it can be observed that the compressive strength of all the foams meets the standard of compressive strength (≥180 kPa) in rigid polyurethane foams for building insulation. Therefore, the potential application of soy oil-based rigid polyurethane foams in the field of building insulation can be illustrated.

**Table 3:** Processing times and mechanical properties of PPUFs and NPUFs

Sample	Apparent density (kg m^−3^)	Compression strength (MPa)	Dimensional stability (%)		
			Length	Width	Thickness
PPUF-0	75.36	0.89	1.32	1.31	0.94
PPUF-10	72.01	0.81	1.06	1.15	0.79
PPUF-20	71.45	0.78	1.18	1.21	0.91
PPUF-30	69.49	0.75	1.34	1.39	0.98
PPUF-40	68.83	0.7	1.54	1.48	1.15
PPUF-50	61.74	0.67	1.76	1.61	1.22
NPUF-0	125.08	1.31	0.97	0.94	0.85
NPUF-10	111.63	1.07	0.87	0.89	0.79
NPUF-20	100.03	1.03	0.73	0.76	0.72
NPUF-30	99.45	0.96	0.58	0.74	0.68
NPUF-40	89.37	0.92	0.52	0.69	0.46
NPUF-50	87.26	0.88	0.43	0.59	0.41

### Dimensional stability

In addition to mechanical strength, dimensional stability is another important characteristic of rigid polyurethane foam used in roofing, insulating, or any other constructing materials. Standard specifications for dimensional stability had been reported to be less than 3% of linear change at 70 °C for 24 h.^41^ From Table 3, we can see that the dimensional changes of all the foams are less than 3% and the dimensional changes of the foams after T–D addition are less than 1%, so the dimensional changes of all the foams meet the standard specifications for dimensional stability.

### Thermogravimetric analysis

**Fig. 8:**
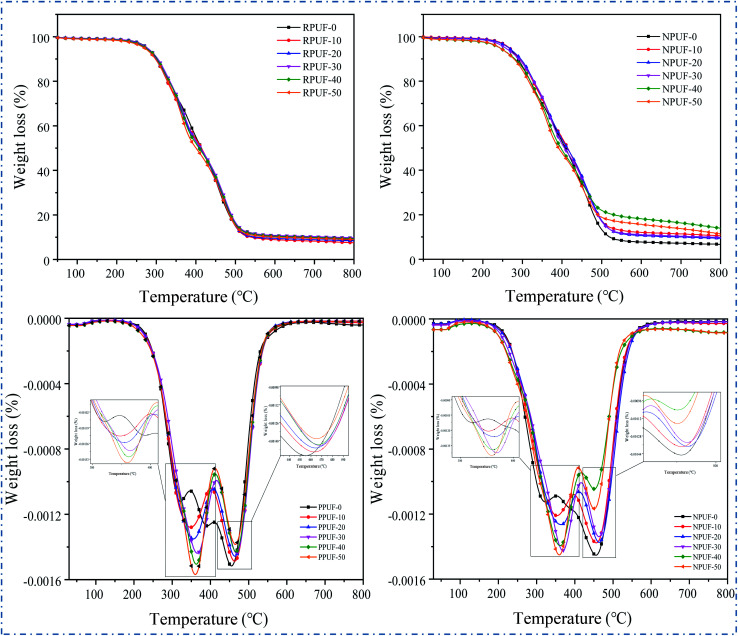
TG and DTG curves of RPUFs and NPUFs.

To evaluate the thermal stability of the prepared PPUFs and NPUFs, TGA is conducted under the flow of nitrogen. The TG and DTG curves of PPUF and NPUF samples are illustrated in Fig. 8, and the representative parameters are summarized in Table 4.

**Table 4:** Thermal properties of PPUFs and NPUFs

Sample	*T*_5%_ (°C)	*T*_50%_ (°C)	*T*_75%_ (°C)	*T*_max1_ (°C)	*T*_max2_ (°C)	Char yield at 800 °C (wt%)
PPUF-0	277	414	472	322	455	8.63
PPUF-10	275	414	474	348	463	7.54
PPUF-20	275	411	474	355	464	8.38
PPUF-30	276	412	478	366	468	9.76
PPUF-40	273	404	476	363	467	9.35
PPUF-50	269	400	473	360	465	8.97
NPUF-0	266	408	469	323	455	6.73
NPUF-10	267	414	476	353	456	10.57
NPUF-20	265	413	479	363	463	9.42
NPUF-30	257	406	476	369	463	9.85
NPUF-40	243	394	481	361	452	13.93
NPUF-50	243	391	471	359	451	11.34

The degradation of foams can be divided into two stages: the degradation in the first stage of weight loss at 300 °C to 350 °C is due to the decomposition of carbamate bonds in the hard segment, and the degradation in the second stage of weight loss at 450 °C to 500 °C is attributed to the thermal degradation in the soft segment.^42^ Conventionally, the thermal stability of RPUFs is described by the temperatures of 5% weight loss (*T*_5%_) considered as the temperature for the onset of degradation.^43^ It can be seen that as the content of flame retardant DOPO–HSBP increased to 50%, the *T*_5%_ of the foam decreased from 277 °C to 269 °C, and the *T*_5%_ of the foam further decreased to 243 °C after the addition of flame retardant T–D. The results showed that the initial decomposition temperature of RPUF showed a decrease after the addition of DOPO–HSBP and T–D. This may be caused by the lower decomposition temperature of DOPO–HSBP and T–D. In addition, it can be seen from *T*_max1_ and *T*_max2_ that the temperature of all PPUFs and NPUFs was increased at *T*_max_, which indicates that the flame retardant will improve the heat endurance of RPUF.

According to the TGA results of the prepared foams, the char residues of the foams prepared with different flame retardants were enhanced compared to the pure RPUF. Noticeably, the char residue of NPUF-40 has the largest increase, reaching 13.93%, which indicates that the addition of DOPO–HSBP and T–D can significantly increase the char residue of RPUF.^44^

### Flame retardancy and combustion behaviors

**Fig. 9:**
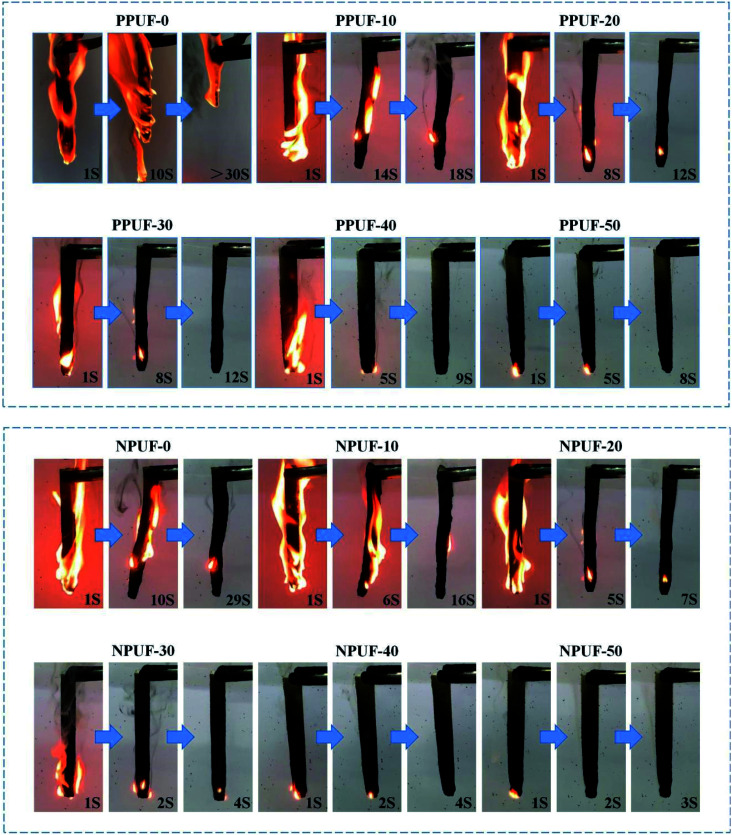
Digital photos of neat PPUFs and NPUFs burned at different times after continuous ignition for 10 s.

To evaluate the flammability of PPUFs and NPUFs, the results of LOI and UL-94 tests for PPUFs and NPUFs with different mass ratios of DOPO–HSBP are presented in Table 5, and digital photos of the vertical burn test (UL-94) are shown in Fig. 9. With the addition of flame retardant DOPO–HSBP, the LOI value of the foam increased from 18.3 to 25.2, and the UL-94 result was classified as “V-0 rating”, which significantly improved the flame retardancy. When flame retardant T–D was added, the possible synergistic effect of phosphorus (DOPO–HSBP) and nitrogen (T–D) could improve the flame retardant activity in the gas-phase and condensed-phase, which increased the LOI value to 25.5 and further improved the flame retardant effect.^45^

**Table 5:** The limiting oxygen index and UL-94 vertical burning test of PPUFs and NPUFs

Sample	LOI (%)	UL-94			
		*t*_1_ a (s)	*t*_2_ a (s)	Dripping	Rating
PPUF-0	18.3	BC^b^	—	Yes	NR^c^
PPUF-10	20.2	19.0	3.7	No	V1
PPUF-20	21.6	12.6	3.1	No	V0
PPUF-30	23.2	10.5	2.9	No	V0
PPUF-40	24.3	8.8	2.5	No	V0
PPUF-50	25.2	7.8	2.1	No	V0
NPUF-0	18.7	29.3	4.0	Yes	V1
NPUF-10	20.8	16.7	3.3	No	V1
NPUF-20	21.9	7.5	3.0	No	V0
NPUF-30	23.7	5.0	2.7	No	V0
NPUF-40	24.6	4.3	2.3	No	V0
NPUF-50	25.5	3.4	2.0	No	V0

a *t*, average combustion times after the flame.

b BC, burns to clamp.

c NR, not rated.

Combined with the UL-94 data in the table and the digital photo figure, it can be seen that the NPUF-50 foam can be self-extinguished and no dripping appears in a short time after burning, which effectively prevents the spread of fire. The formation of a char can be observed on the surface after combustion, which can effectively prevent heat and oxygen from diffusing into the internal matrix of the polymer and improve the flame retardancy of the foam.^46^ All of the above revealed the addition of DOPO–HSBP and T–D is beneficial to improve the flame retardant properties of the foam.

## Conclusions

In this study, a phosphorus-containing bio-based polyol DOPO–HSBP and a nitrogen-containing polyol T–D were synthesized and added as flame retardants to an environmentally friendly soy oil-based rigid polyurethane foam prepared from soybean oil. It was observed that the synergistic effect of phosphorus and nitrogen of flame retardants DOPO–HSBP and T–D greatly improved the flame retardancy of RPUF by LOI and UL-94, and the LOI value increased from 18.3 to 25.5, and the UL-94 grade was improved to “V0”, this may be because DOPO–HSBP and T–D are decomposed from the foam at a lower temperature, thus preventing further burning of the foam earlier and more efficiently. The compressive strength and SEM of the foam showed that both the compressive strength and the size of the pores of the NPUF-50 sample were almost unchanged compared to the polyurethane rigid foam without flame retardant. Therefore, with the addition of flame retardants DOPO–HSBP and T–D in appropriate amounts, it can not only greatly improve the flame retardant properties of the foam but also has little effect on the mechanical properties of the foam. Moreover, the compressive strength and dimensional stability of rigid polyurethane foam prepared from bio-based polyols meet the standards of construction and other fields. From the TG and DTG data of the foams, it can be observed that the addition of DOPO–HSBP and T–D greatly improved the *T*_max_ and carbon residue of the foams, which also confirms the improvement of the flame retardant properties of the foams by DOPO–HSBP and T–D. Therefore, it can be seen that the phosphorus and nitrogen synergistic flame retardant system for foam flame retardant performance is much higher than the phosphorus flame retardant system alone. This research would help us to further research and develop novel bio-based RPUF materials with excellent flame-retardant effects.

## Conflicts of interest

There are no conflicts to declare.

## Supplementary Material

## References

[cit1] Auras R. A. , Lim L. T., Selke S. E. M. and Tsuji H., Poly(Lactic Acid): Synthesis, Structures, Properties, Processing, and Applications, John Wiley & Sons, New Jersey, 1st edn, 2010

[cit2] Guo C., Guo X., Cai N., Dong Y. (2012). Novel fabrication method of porous poly(lactic acid) scaffold with hydroxyapatite coating. Mater. Lett..

[cit3] Yao R., Yao Z., Zhou J., Zhou C. (2016). Microcosmic morphology and properties of hollow glass beads-reinforced polylactic acid-based foam composites. Polym. Compos..

[cit4] Fang Y. S. and Zhu L. M., Polyurethane foam, Chemical Industry Press, Beijing, 2005

[cit5] Zhou Y., Bu R., Gong J., Yan W., Fan C. (2018). Experimental investigation on downward flame spread over rigid polyurethane and extruded polystyrene foams. Exp. Therm. Fluid Sci..

[cit6] Chattopadhyay D. K., Webster D. C. (2009). Thermal stability and flame retardancy of polyurethanes. Prog. Polym. Sci..

[cit7] Bhoyate S., Ionescu M., Radojcic D., Kahol P. K., Chen J., Mishra S. R., Gupta R. K. (2018). Highly flame-retardant bio-based polyurethanes using novel reactive polyols. J. Appl. Polym. Sci..

[cit8] Michalowski S., Hebda E., Pielichowski K. (2017). Thermal stability and flammability of polyurethane foams chemically reinforced at POSS. J. Therm. Anal. Calorim..

[cit9] Wang Y., Feng W., Dong Q., Xie M., Peng L., Ding Y., Zhang S., Yang M., Zheng G. Q. (2017). Core–shell expandable graphite@aluminum hydroxide as a flame-retardant for rigid polyurethane foams. Polym. Degrad. Stab..

[cit10] Duan H. J., Kang H. Q., Zhang W. Q., Ji X., Li Z. M., Tang J. H. (2013). Core–shell structure design of pulverized expandable graphite particles and their application in flame-retardant rigid polyurethane foams. Polym. Int..

[cit11] Xu W., Wang G., Zheng X. (2015). Research on Highly Flame-retardant Rigid PU Foams by Combination of Nanostructured Additives and Phosphorus Flame Retardants. Polym. Degrad. Stab..

[cit12] Xu Q., Zhai H., Wang G. (2015). Mechanism of smoke suppression by melamine in rigid polyurethane foam. Fire Mater..

[cit13] Zhu H., Xu S.-a. (2018). Preparation and fire behavior of rigid polyurethane foams synthesized from modified urea–melamine–formaldehyde resins. RSC Adv..

[cit14] Li Q., Wang J., Chen L., Shi H., Hao J. (2019). Ammonium polyphosphate modified with β-cyclodextrin crosslinking rigid polyurethane foam: Enhancing thermal stability and suppressing flame spread. Polym. Degrad. Stab..

[cit15] Chen H. B., Shen P., Chen M. (2016). *et al.*, Highly Efficient Flame Retardant Polyurethane Foam with Alginate/Clay Aerogel Coating. ACS Appl. Mater. Interfaces.

[cit16] Luo F., Wu K., Li Y., Jian Z., Guo H., Lu M. (2015). Reactive flame retardant with core–shell structure and its flame retardancy in rigid polyurethane foam. J. Appl. Polym. Sci..

[cit17] Rong Y., Hu W., Liang X., Yan S., Li J. C. (2015). Synthesis, mechanical properties and fire behaviors of rigid polyurethane foam with a reactive flame retardant containing phosphazene and phosphate. Polym. Degrad. Stab..

[cit18] Yang R., Wang B., Han X., Ma B., Li J. (2017). Synthesis and characterization of flame retardant rigid polyurethane
foam based on a reactive flame retardant containing phosphazene and cyclophosphonate. Polym. Degrad. Stab..

[cit19] Wang S. X., Zhao H. B., Rao W. H., Huang S. C., Wang T., Liao W., Wang Y. Z. (2018). Inherently flame-retardant rigid polyurethane foams with excellent thermal insulation and mechanical properties. Polymer.

[cit20] Ciesielski M., Schaefer A., Doering M. (2008). Novel efficient DOPO-based flame-retardants for PWB relevant epoxy resins with high glass transition temperatures. Polym. Adv. Technol..

[cit21] Liu Y. L., Chang G. P., Wu C. S. (2006). Halogen-free flame retardant epoxy resins from hybrids of phosphorus- or silicon-containing epoxies with an amine resin. J. Appl. Polym. Sci..

[cit22] Wang J., Xu B., Wang X., Liu Y. (2021). A phosphorous-based bi-functional flame retardant for rigid polyurethane foam. Polym. Degrad. Stab..

[cit23] Gaan S., Sun G., Hutches K., Engelhard M. H. (2008). Effect of nitrogen additives on flame retardant action of tributyl phosphate: phosphorus–nitrogen synergism. Polym. Degrad. Stab..

[cit24] Silva V. R., Mosiewicki M. A., Yoshida M. I., Silva M. C., Stefani P. M., Marcovich N. E. (2013). Polyurethane foams based on modified tung oil and reinforced with rice husk ash II: mechanical characterization. Polym. Test..

[cit25] Veronese V. B., Menger R. K., Forte M. M. D., Petzhold C. L. (2011). Rigid polyurethane foam based on modified vegetable oil. J. Appl. Polym. Sci..

[cit26] Guo A., Javni I., Petrovic Z. (2000). Rigid polyurethane foams based on soybean oil. J. Appl. Polym. Sci..

[cit27] Ji D., Fang Z., He W., Luo Z., Jiang X., Wang T., Guo K. (2015). Polyurethane rigid foams formed from different soy-based polyols by the ring opening of epoxidized soybean oil with methanol, phenol, and cyclohexanol. Ind. Crops Prod..

[cit28] Klaas M. R., Warwel S. (1999). Complete and partial epoxidation of plant oils by lipase-catalyzed perhydrolysis. Ind. Crops Prod..

[cit29] Ahn B. K., Kraft S., Wang D., Sun X. S. (2011). Thermally Stable, Transparent, Pressure-Sensitive Adhesives from Epoxidized and Dihydroxyl Soybean Oil. Biomacromolecules.

[cit30] Pillai P. K. S., Li S., Bouzidi L., Narine S. S. (2016). Solvent-free synthesis of polyols from 1-butene metathesized palm oil for use in polyurethane foams. J. Appl. Polym. Sci..

[cit31] Ahn B. K., Sung J., Kim N., Kraft S., Sun X. S. (2013). UV-curable pressure-sensitive adhesives derived from functionalized soybean oils and rosin ester. Polym. Int..

[cit32] Liu P., Liu M., Gao C. (2013). *et al.*, Preparation, characterization and properties of a halogen-free phosphorous flame-retarded poly(butylene terephthalate) composite based on a DOPO derivative. J. Appl. Polym. Sci..

[cit33] Salih S. M. , Fourier Transform: Materials Analysis, Intechopen, London, UK, 1st edn, 2012

[cit34] Eisa M. Y., Dabbas M. A., Abdulla F. H. (2015). Quantitative identification of phosphate using X-ray diffraction and Fourier transform infrared (FTIR) spectroscopy. Int. J. Curr. Microbiol. Appl. Sci..

[cit35] Jia P., Zhang M., Hu L. (2015). *et al.*, Synthesis and application of phosphaphenanthrene groups-containing soybean-oil-based plasticizer. Ind. Crops Prod..

[cit36] Chen X., Hu Y., Jiao C., Song L. (2007). Preparation and thermal properties of a novel flame-retardant coating. Polym. Degrad. Stab..

[cit37] Qian X., Song L., Jiang S., Tang G., Xing W., Wang B., Hu Y., Yuen R. K. K. (2013). Novel Flame Retardants Containing 9,10-Dihydro-9-oxa-10-phosphaphenanthrene-10-oxide and Unsaturated Bonds: Synthesis, Characterization, and Application in the Flame Retardancy of Epoxy Acrylates. Ind. Eng. Chem. Res..

[cit38] Zieleniewska M. (2015). *et al.*, Preparation and characterization of rigid polyurethane foams using a rapeseed oil-based polyol. Ind. Crops Prod..

[cit39] Cheng J., Ma D., Li S. (2020). *et al.*, Preparation of zeolitic imidazolate frameworks and their application as flame retardant and smoke suppression agent for rigid polyurethane foams. Polymers.

[cit40] Hebda E., Bukowczan A., Ozimek J. (2018). *et al.*, Rigid polyurethane foams reinforced with disilanolisobutyl POSS: Synthesis and properties. Polym. Adv. Technol..

[cit41] Ji D., Fang Z., He W. (2015). *et al.*, Synthesis of soy-polyols using a continuous microflow system and preparation of soy-based polyurethane rigid foams. ACS Sustainable Chem. Eng..

[cit42] Wang S. X., Zhao H. B., Rao W. H., Huang S. C., Wang T., Liao W., Wang Y. Z. (2018). Inherently flame-retardant rigid polyurethane foams with excellent thermal insulation and mechanical properties. Polymer.

[cit43] Zhang M., Luo Z., Zhang J., Chen S., Zhou Y. (2015). Effects of a novel phosphorus–nitrogen flame retardant on rosin-based rigid polyurethane foams. Polym. Degrad. Stab..

[cit44] Xu W., Wang G., Xu J. (2019). *et al.*, Modification of diatomite with melamine coated zeolitic imidazolate framework-8 as an effective flame retardant to enhance flame retardancy and smoke suppression of rigid polyurethane foam. J. Hazard. Mater..

[cit45] Zhang M., Luo Z., Zhang J., Chen S., Zhou Y. (2015). Effects of a novel phosphorus–nitrogen flame retardant on rosin-based rigid polyurethane foams. Polym. Degrad. Stab..

[cit46] Tsa B., Wpa B. (2020). Fire-extinguishing characteristics and flame retardant mechanism of polylactide foams: Influence of tricresyl phosphate combined with natural flame retardant. Int. J. Biol. Macromol..

